# Ultrasound-assisted structural characterization and properties of glycosylation-modified products of the British red kidney bean protein antioxidant peptide fractions

**DOI:** 10.3389/fnut.2025.1577660

**Published:** 2025-06-10

**Authors:** Xiaoyu Fan, Yurui Li, Suping Cui

**Affiliations:** ^1^College of Food Science in Heilongjiang Bayi Agricultural University, Daqing, China; ^2^National Coarse Cereals Engineering Research Center, Daqing, China

**Keywords:** antioxidant peptides, *in vitro* antioxidant activity, ultrasound-assisted glycosylation, structural properties, functional properties

## Abstract

**Introduction:**

In order to clarify the effects of glycosylation reaction under ultrasonic conditions on the *in vitro* antioxidant activity, structural properties and functional properties of the British red kidney bean protein antioxidant peptide fractions, the British red kidney bean protein antioxidant peptide fractions and glucose were used as raw materials.

**Methods:**

Four experimental groups were established: BHPs, US, GR, and US-GR. The study investigated the following characteristics among the treatment groups: grafting degree, browning degree, antioxidant activities (total antioxidant capacity, hydroxyl radical scavenging activity, DPPH radical scavenging activity, superoxide anion scavenging activity, reducing power), physicochemical properties (Free amino group content, Total and free sulfhydryl group content), structural characterization (atomic force microscopy (AFM), intrinsic fluorescence spectroscopy, surface hydrophobicity, circular dichroism spectroscopy (CD), fourier transform infrared spectroscopy (FTIR)), functional properties(solubility, foaming properties, emulsifying properties).

**Results:**

The results showed that the antioxidant activities of the ultrasound-assisted glycosylation products were significantly enhanced (*P* < 0.05) compared with those of the British red kidney bean protein antioxidant peptide fractions, with the greatest enhancement in reducing power (105.38%) and the smallest enhancement in the scavenging rate of superoxide anion radical scavenging activity (32.35%); the ultrasound-assisted glycosylation resulted in an increase in the degree of grafting by 36.16%, decreased free amino acid content by 33.58%, decreased total sulfhydryl content, increased free sulfhydryl groups, smaller 3D size distribution on mesoscopic scale, decreased endogenous fluorescence intensity and surface hydrophobicity, and decreased β-sheet content and increased random coils content.

**Discussion:**

These results suggest that ultrasound promoted the interaction between the British red kidney bean protein antioxidant peptide fractions and the glucose glycosylation reaction. The products of ultrasound-assisted glycosylation had higher solubility, foaming and emulsification properties, which significantly improved the functional properties of the glycosylated products. The present research provides a basis for the ultrasound-assisted glycosylation of the British red kidney bean protein antioxidant peptide fractions.

## Introduction

Antioxidant peptides are bioactive components primarily obtained by hydrolyzing proteins from animals, plants, or microorganisms. These peptides prevent oxidative damage through various mechanisms and present several advantages over synthetic antioxidants, including non-toxicity, easy absorption by the human body, and suitability as functional food ingredients or dietary supplements (1). However, their antioxidant efficacy is often lower than that of synthetic alternatives, which limits their broader, high-value applications. Improving the antioxidant potential of these peptides through effective modification strategies creates opportunities for their enhanced use in various fields.

Protein and peptide modification techniques are generally categorized into chemical, physical, and enzymatic methods. Chemical modifications include processes such as glycosylation, phosphorylation, acylation, deamidation, covalent crosslinking, hydrolysis, and oxidation (2). Glycosylation Reaction (GR), a non-enzymatic browning process, involves the covalent bonding of amino groups in amino acids with the carbonyl groups of reducing sugars. This method is a natural and non-toxic means of modifying proteins (or peptides), significantly altering their physiological activities and functional properties (3). For example, glycosylation has been shown to enhance the antioxidant activity, solubility, and foaming properties of zein and whey protein peptides (4, 5). However, conventional heat-induced glycosylation may generate undesirable byproducts, such as furans and heterocyclic amines, during later stages of processing, which can negatively impact product quality and flavor (6). To mitigate the formation of these harmful substances, non-thermal methods for inducing and accelerating peptide glycosylation are being explored. Ultrasound technology (US), an emerging non-thermal technique, improves peptide functionality through mechanisms like thermal effects, acoustic cavitation, acoustic streaming, and particle oscillation (7). Research has shown that ultrasound-assisted glycosylation (US-GR) can further enhance the physiological properties of food-derived proteins and peptides. For example, Wang et al. (8) demonstrated that ultrasound-assisted glycosylation improved both the antioxidant activity and functional properties of hydrolysates from golden threadfin bream muscle proteins. Similarly, Aziznia et al. (9) used ultrasound to facilitate the glycosylation of mung bean protein isolate with maltodextrin, enhancing its solubility and emulsifying capacity.

British red kidney beans contain approximately 22.3% protein, making them an excellent source of high-quality protein peptides. Research has indicated that a high proportion of hydrophobic amino acids is a critical structural feature of protein peptides (10). In previous studies conducted by our research team, the British red kidney bean protein (BHP) was hydrolyzed using alkaline protease to obtain antioxidant peptides (BHPs). Cell and animal experiments demonstrated their notable antioxidant activity (11, 12). Moreover, glycosylation was shown to further enhance their activity (2), although it still falls short compared to synthetic antioxidants and common natural antioxidants, such as vitamin C (VC) and vitamin E (VE) (13). In this research, the optimal ultrasound-assisted glycosylation conditions for the British red kidney bean protein antioxidant peptides were obtained through one-factor and response surface optimization tests in the early stage (Ultrasound-assisted glycosylation modification conditions: ultrasound power 145 W, ultrasound frequency 40 kHz, ultrasound temperature 68°C, pH 9, ultrasound time 57 min), and the antioxidant capacity of the modified products was improved, but the structural features and functional properties of the modified products have not yet been clarified. Therefore, this research is intended to provide a theoretical basis and technical guidance for the development and utilization of related products by characterizing the structural and functional characteristics of the modified products.

## Materials and methods

### Materials

British red kidney beans were purchased from Daqing Xinmate Supermarket. Alkaline protease (2 × 105 U/g) and ascorbic acid were obtained from Beijing Solarbio Science & Technology Co., Ltd. Total antioxidant capacity detection kit, hydroxyl radical scavenging rate detection kit, and DPPH radical scavenging rate detection kit were provided by Nanjing Jiancheng Bioengineering Institute. All other reagents were of analytical grade.

### Preparation of British red kidney bean protein antioxidant peptide fractions

The extraction of British red kidney bean protein (BHP) and preparation of its antioxidant peptide fractions (BHPs) followed the method described by Gao et al. (14).

### Preparation of glycosylation products

One gram of the British red kidney bean protein antioxidant peptide fractions was dissolved in 40 mL of distilled water, and 12.5 mg/mL glucose solution was prepared. These solutions were used to create a peptide solution with a concentration of 2.5% (w/v) and a sugar-to-peptide ratio of 1:2 for the glycopeptide mixture. Ultrasound modification group (US): The peptide solution was adjusted to pH 9 and subjected to ultrasonic treatment at 145 W, 40 kHz, and 68°C for 57 min. The resulting solution was freeze-dried for later use. Glycosylation modification group (GR): The glycopeptide mixture was incubated in a water bath at 68°C and pH 9 for 57 minutes. The solution was then freeze-dried for later use. Ultrasound-assisted glycosylation modification group (US-GR): The glycopeptide mixture was treated under ultrasonic conditions at 145 W, 40 kHz, and 68°C with pH adjusted to 9 for 57 minutes. The resulting solution was freeze-dried for later use.

### Determination of grafting degree and browning degree

The method was adapted from the TNBS (trinitrobenzenesulfonic acid) assay described by Xu et al. (15). Freeze-dried modified products were dissolved in 50 mmol/L phosphate buffer to prepare 0.1% (w/v) solution. To initiate the reaction, 1 mL of 0.1% (w/v) TNBS solution was added to 1 mL of the modified product solution and incubated in the dark for 2 h. The reaction was terminated by adding 1 mL of 1% (w/v) SDS and 0.5 mL of 1 mol/L HCl. 200 μL aliquot of the reaction mixture was then measured at 335 nm using a microplate reader (denoted as S_0_ before the reaction and S_1_ after the reaction). The grafting degree was calculated using the formula provided in Equation 1.


(1) $$\begin{array}{l} Grafting\;degree\;\left(\%\right)=\frac{S_{0}-S_{1}}{S_{0}}\times 100 \end{array}$$

The browning degree of the modified products was determined following the method of Zhang et al. (16). The absorbance of 0.1% (w/v) modified product solution was measured at 420 nm using a UV spectrophotometer. Double-distilled water was used for baseline calibration prior to measurement.

### *In vitro* antioxidant activity

Samples (0.1 g) were dissolved in 10 mL distilled water to prepare 1% (w/v) solution, and VC solution (1%, w/v) was used as a control. Total antioxidant capacity, hydroxyl radical scavenging activity, and DPPH radical scavenging activity were determined using commercial kits (Nanjing Jiancheng Bioengineering Institute). Superoxide anion scavenging activity and reducing power were determined following the method described by Sun et al. (17).

### Physicochemical property

#### Determination of free amino group content

The free amino acid content of the reaction products was determined using the OPA (o-phthaldialdehyde) method as described by Wu et al. (18).

#### Determination of total and free sulfhydryl content

The total and free sulfhydryl content was determined using the method described by Sheng et al. (19). To this end, 0.1 mL of 0.1% (w/v) modified product solution was combined with 0.9 mL of Tris-HCl buffer (50 mmol/L, pH 8.5) containing 0.5% (w/v) SDS, and then 0.02 mL of 10 mmol/L DTNB [5,5′-dithiobis(2-nitrobenzoic acid)] solution was added. The mixture was allowed to react at room temperature for 5 min, and the absorbance at 412 nm was measured using a UV spectrophotometer. The molar extinction coefficient of 1.36 × 10^−^4 M^−1^ cm^−1^ was used to calculate the total thiol content per gram of the sample. For free thiol content determination, the same procedure was followed except the Tris-HCl buffer did not contain SDS.

### Structural characterization of glycosylation products

#### Atomic force microscopy observation

The aggregation state of each group of samples was determined using atomic force microscopy (AFM) following the method of Xu et al. (20). 0.01 g/L modified product solution was prepared in deionized water. 10 μL aliquot was deposited on the surface of freshly cleaved mica (~1.0 × 1.0 cm^2^) and air-dried before imaging with AFM.

#### Intrinsic fluorescence spectroscopy

Intrinsic fluorescence spectra of the modified products were analyzed using a fluorescence spectrophotometer as described by Liu et al. (21). 0.03 mg/mL modified product solution was prepared in 0.01 mol/L phosphate buffer (pH 7.0). Fluorescence absorption was measured within the 300–500 nm wavelength range with an excitation wavelength of 280 nm, slit width of 10 nm, and scanning speed of 1,200 nm/min.

#### Surface hydrophobicity (*H*_0_)

Surface hydrophobicity of the modified products was measured using 1-anilino-8-naphthalene sulfonate (ANS) as a hydrophobic fluorescence probe, following the method of Li et al. (22) with slight modifications. 4 mL sample of modified product solution (0.1 mg/mL) was mixed with 20 μL of ANS solution (8 mmol/L). Fluorescence was measured using a fluorescence spectrophotometer at 25°C with an excitation wavelength of 375 nm, emission wavelength range of 410–600 nm, and a scan speed of 10 nm/min.

#### Circular dichroism (CD) spectroscopy

Circular dichroism (CD) spectroscopy was used to determine changes in the secondary structure of the modified products, following the method of Li et al. (23) with slight modifications. The samples were dissolved in PBS (pH 7.4) to prepare 0.2 mg/mL solution. CD spectra were measured in the range of 190–260 nm at 25°C. The scan rate and resolution were set to 75 nm/min and 0.1 nm, respectively. Data were processed using the CDNN software, and the content and proportion of the secondary structure were calculated.

#### Fourier-transform infrared (FTIR) spectroscopy

FTIR infrared spectroscopy was performed as described by Zhang et al. (24) with minor modifications. 2.0 mg sample of the modified product was mixed with dried potassium bromide powder at a 1:100 mass ratio, and the mixture was ground into a uniform powder for pellet formation. FTIR spectra were obtained using a Fourier-transform infrared spectrometer. The scanning parameters were set as follows: frequency range 4,000–400 cm^1^, resolution 4 cm^1^, and 32 scans.

### Functional properties of glycosylation products

#### Solubility

Solubility of the modified products was determined with slight modifications to the method of Kang et al. (25). 10 mg/mL solution of the modified product was prepared and the pH was adjusted to 7.0. The solution was then stirred at 20°C for 30 min using a magnetic stirrer, followed by centrifugation at 8,600 × g for 20 min. The supernatant was collected and stored at 4°C.

#### Foam properties

Foam ability (FA) and foam stability (FS) were measured using a modified method from Li et al. (26). 0.2 g aliquot of freeze-dried modified product was dispersed into 20 mL of distilled water and processed at room temperature for 1 min at 10,000 rpm using a rotary rheometer. The solution was then transferred to a 100 mL graduated cylinder. The initial volume (V_0_), foam volume after 30 s of stirring (V_1_), and foam volume after 30 min of storage at room temperature (V_30_) were recorded. FA and FS were calculated using the following formulas:


(2) $$\begin{array}{l} FA\left(\%\right)=\frac{V_{1}}{V_{0}}\times 100 \end{array}$$

(3) $$\begin{array}{l} FS\left(\%\right)=\frac{V_{30}}{V_{1}}\times 100 \end{array}$$

#### Emulsification properties

The emulsifying activity index (EAI) and emulsifying stability index (ESI) of the glycosylated products were determined following the method of Pi et al. (27). 150 mL solution of the modified product (0.1%, w/v, in 10 mmol/L phosphate buffer, pH 7.0) was mixed with 50 mL soybean oil. The mixture was homogenized at 12,000 rpm for 1 min to form a stable emulsion. Immediately after, 50 μL of the emulsion from the bottom was extracted and dispersed into 5 mL of 0.1% (w/v) SDS solution. The absorbance at 650 nm (A_0_) was measured immediately, and the procedure was repeated to measure absorbance (A_10_) after a 10-min interval. The formulas for calculating EAI and ESI are as follows:


(4) $$\begin{array}{l} EAI\left(\frac{m^{2}}{g}\right)=\frac{2\times 2.303\times A_{0}\times n}{\rho \times \varphi \times 10^{4}} \end{array}$$

(5) $$\begin{array}{l} ESI\left(\%\right)=\frac{A_{10}}{A_{0}} \end{array}$$

Where ρ is the sample concentration (g/L); ϕ is the oil phase volume fraction, n is the dilution factor.

### Statistical analysis

All experiments were performed in triplicate, and the mean values were taken. Statistical and variance analyses (ANOVA) were conducted using SPSS 25.0 software. Duncan's method was used for significant difference analysis (*p* < 0.05). Related graphs and charts were prepared using Origin 8.0 software.

## Results and discussion

### Analysis of degree of grafting and browning intensity

The degree of grafting and browning intensity are commonly used to evaluate the extent of glycosylation reactions (28).Glycosylation reactions occurred in both the glycosylation modification group (US) and the ultrasound-assisted glycosylation modification group (US-GR), with grafting degrees of 26.27% and 35.77%, respectively (Table 1). The degree of grafting in the US-GR group was significantly higher than that in the US group (*p* < 0.05), indicating that ultrasound promotes glycosylation reactions between the British red kidney bean protein antioxidant peptide fractions and glucose. Additionally, the browning intensity of the US-GR group was significantly higher than that of the BHPs, US, and GR groups (*p* < 0.05), suggesting that the glycosylation extent was highest in the US-GR group. Chen et al. (29) used ultrasound-assisted glycosylation to prepare high solubility rice proteins and showed that the grafting degree of the ultrasound-assisted glycosylation product was 2.08 times higher than that of the conventional glycosylation product. Research has shown that ultrasound enhances the effective collisions between proteins and reducing sugars, thereby facilitating glycosylation reactions between proteins (or peptides) and carbohydrates (30). Moreover, the thermal effect induced by the cavitation effect of ultrasound reduces the activation energy required for intermediates and melanoidin formation in glycosylation reactions, leading to increased browning intensity (31).

**Table 1:** Changes in the degree of grafting and browning before and after ultrasound-assisted glycosylation modification of the British red kidney bean protein antioxidant peptide fractions.

**Sample**	**Grafting degree (%)**	**Browning degree (A420 absorbance value)**
BHPs	——	0.093 ± 0.013^c^
US	——	0.098 ± 0.011^c^
GR	26.27 ± 0.22^b^	0.357 ± 0.015^b^
US-GR	35.77 ± 0.16^a^	0.412 ± 0.011^a^

Different lowercase letters over column indicate significant differences (p < 0.05).

### Analysis of *in vitro* antioxidant activity

**Figure 1:**
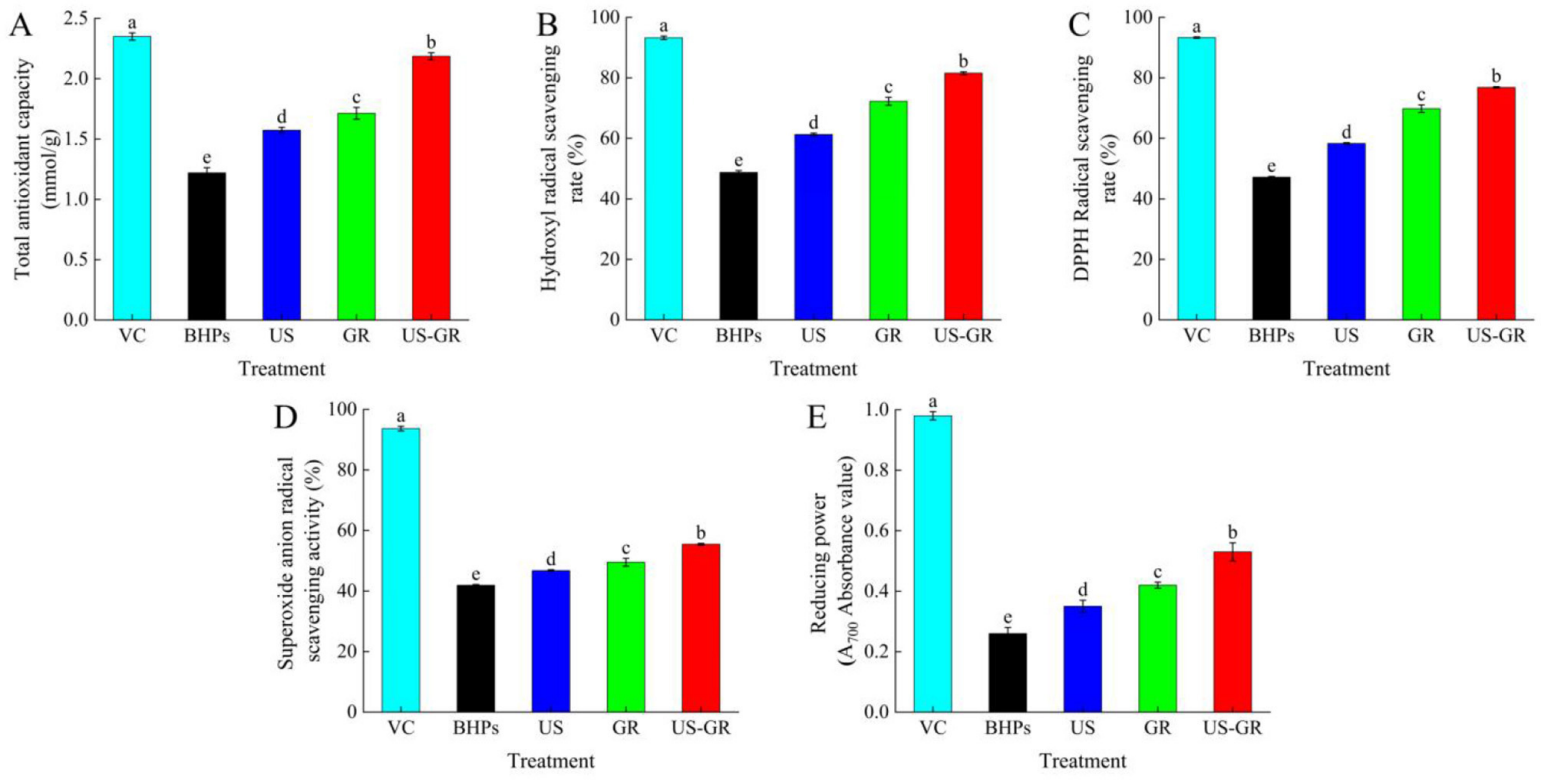
Changes in total antioxidant capacity **(A)**, hydroxyl radical scavenging rate **(B)**, DPPH radical scavenging rate **(C)**, superoxide anion radical scavenging activity **(D)** and reducing power **(E)** of the British red kidney bean protein antioxidant peptide fractions before and after ultrasound-assisted glycosylation modification. Different lowercase letters over column indicate significant differences (*p* < 0.05).

Antioxidants typically act as free radical scavengers, singlet oxygen quenchers, and metal ion chelators, among other roles (30). As shown in Figure 1, the *in vitro* antioxidant activities of the British red kidney bean protein antioxidant peptide fractions (BHPs) were significantly enhanced (*p* < 0.05) after single ultrasound (US) or glycosylation (GR) treatment. Compared with the US or GR treatment groups, the US-GR modification resulted in a significant enhancement of all antioxidant activity indices (*p* < 0.05), with the most substantial improvement observed in reducing power (105.38%). Ultrasound-assisted glycosylation further narrows the gap between the *in vitro* antioxidant activities of the British red kidney bean protein antioxidant peptide fractions and VC. This demonstrates that US-GR treatment effectively enhances the antioxidant activity of BHPs. Current studies indicate that ultrasound contributes to the unfolding of protein peptide structures, exposing more binding sites for free radicals, which not only enhances the activity of antioxidant peptides but also facilitates glycosylation by exposing glycosylation sites and promoting the reaction. The introduction of hydroxyl-rich side chains provides more protons, enabling the resulting glycosylation products to better scavenge free radicals (4, 32). In summary, US-GR treatment is a feasible and effective method to improve the antioxidant activity of the British red kidney bean protein antioxidant peptide fractions.

### Physicochemical property analysis

#### Determination of free amino group content

**Figure 2:**
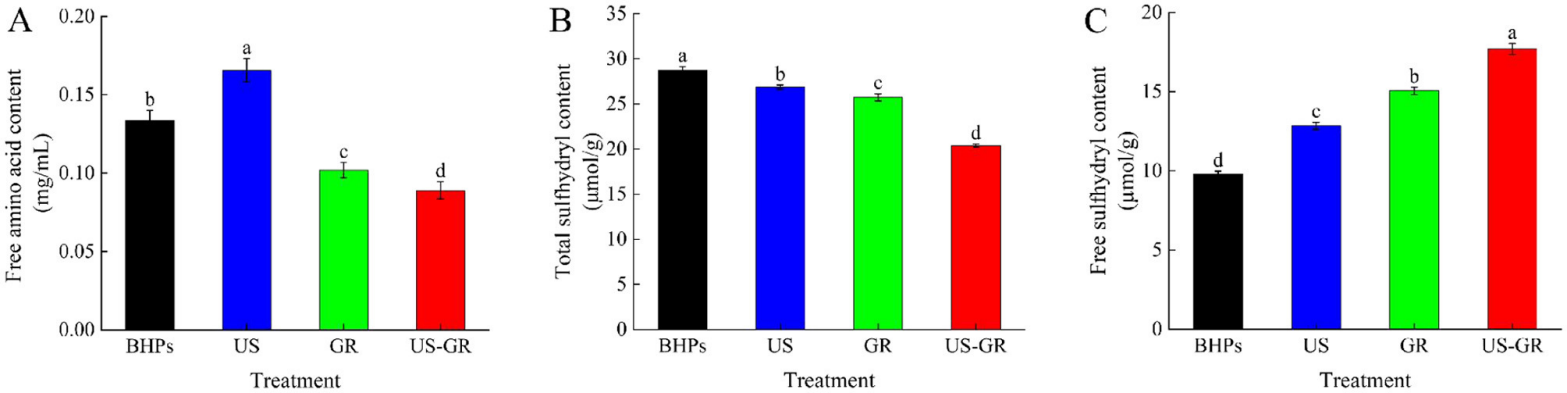
Changes in free amino acid content **(A)**, total sulfhydryl content **(B)** and free sulfhydryl content **(C)** of the British red kidney bean protein antioxidant peptide fractions before and after ultrasound-assisted glycosylation modification. Different lowercase letters over column indicate significant differences (*p* < 0.05).

Protein glycosylation is a non-enzymatic browning reaction in which the amino groups of proteins covalently bind to the carbonyl groups of sugars, resulting in a reduction in the free amino content of the protein. Therefore, free amino content is another critical indicator for evaluating the extent of protein glycosylation reactions (16). As shown in Figure 2A, the free amino acid content of British red kidney bean protein peptides (BHPs) was 0.1336 mg/mL. After ultrasound treatment (US), the content significantly increased to 0.1656 mg/mL. Glycosylation treatment (GR) significantly reduced it to 0.1022 mg/mL. Furthermore, the free amino acid content of the ultrasound-assisted glycosylation modification product (US-GR) decreased even further compared to the GR group (*p* < 0.05). The results indicate that glycosylation modification causes a reaction between free amino acids and the carbonyl groups of reducing sugars, leading to a decrease in free amino acid content (33). Ultrasound treatment facilitates the exposure of amino acids in peptide components, increasing the effective collisions between peptides and reducing sugars (30), thereby promoting glycosylation reactions between the carbonyl groups of glucose and the antioxidant peptides of the British red kidney bean protein antioxidant peptide fractions, ultimately leading to a decrease in free amino content.

#### Determination of total and free sulfhydryl content

Changes in total and free sulfhydryl content reflect the spatial extension of modified molecules during glycosylation reactions (34). As shown in Figures 2B, C, the total sulfhydryl content of the BHPs, US, GR, and US-GR groups was 28.77 μmol/g, 26.86 μmol/g, 25.74 μmol/g, and 20.39 μmol/g, respectively, while the free sulfhydryl content was 9.80 μmol/g, 12.84 μmol/g, 15.05 μmol/g, and 17.70 μmol/g, respectively. Compared to BHPs, the US, GR, and US-GR treatments resulted in a significant decrease in total sulfhydryl content and a significant increase in free sulfhydryl content (*p* < 0.05). Among these, the US-GR treatment group exhibited the most pronounced changes, with significant differences. The cavitation effect of ultrasound readily causes the breakage of covalent bonds between peptide fragments, promoting structural unfolding of peptides and exposing -SH groups (35). Additionally, the covalent binding of the glycosylation reaction (GR) facilitates structural unfolding of the peptides, breaking disulfide bonds linked between chains, thereby reducing total sulfhydryl content while increasing free sulfhydryl content (36). These findings indicate that ultrasound-assisted glycosylation treatment (US-GR) significantly affects the total and free sulfhydryl content of peptide components (BHPs), further confirming that ultrasound (US) promotes the glycosylation reaction of BHPs. These results are consistent with those reported by Zhao et al. (31).

### Structural characterization analysis

#### Atomic force microscopy

**Figure 3:**
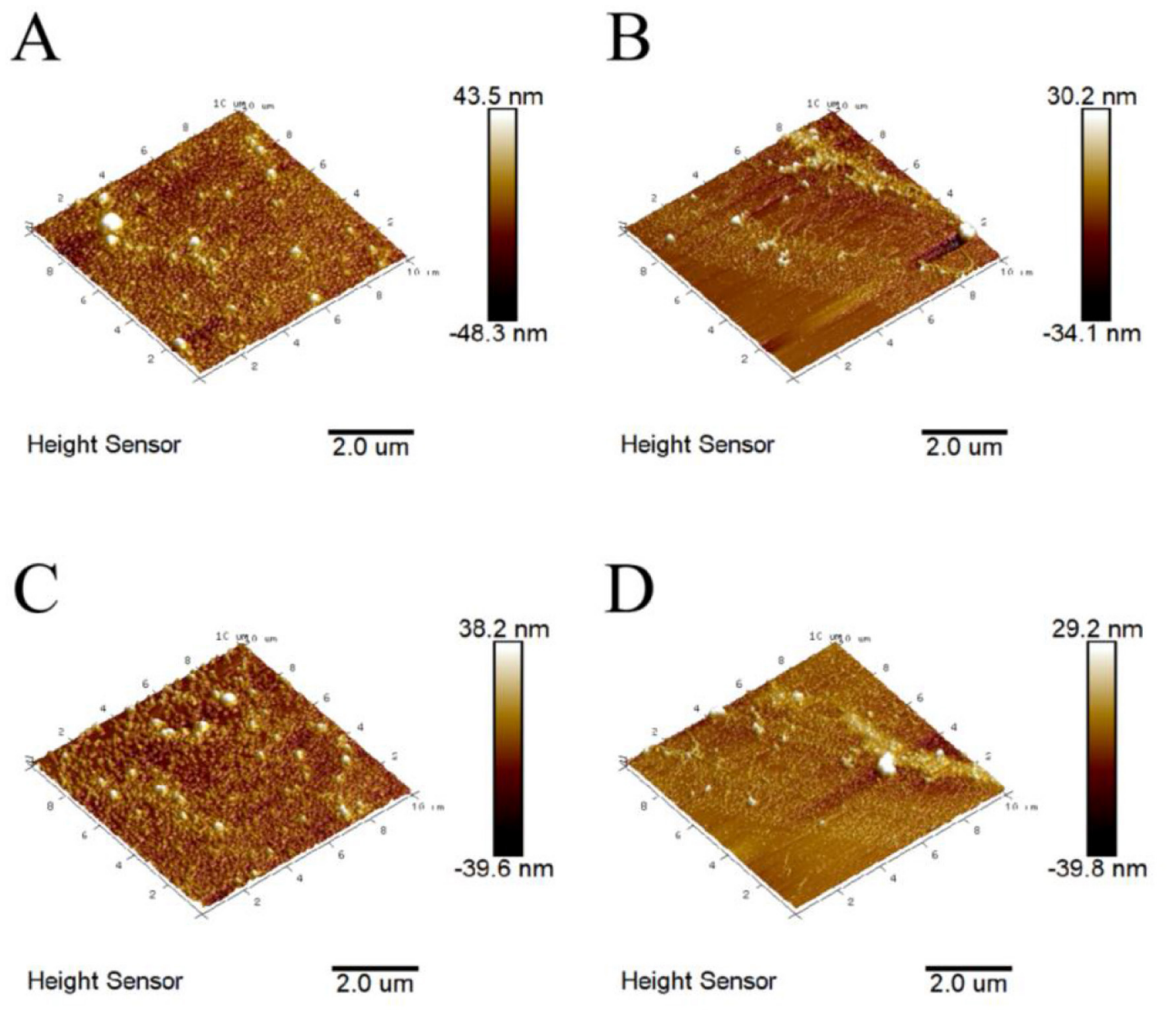
Atomic force microscopy images before and after ultrasound-assisted glycosylation modification of the British red kidney bean protein antioxidant peptide fractions. **(A–D)** represent the three-dimensional distribution maps of British red kidney bean protein antioxidant peptide components (BHPs), ultrasound-treated group (US), glycosylation-modified group (GR), and ultrasound-assisted glycosylation-modified group (US-GR), respectively.

Atomic force microscopy (AFM) is commonly used to characterize the three-dimensional morphology of substances (37). The three-dimensional size distribution range of BHPs was found to be −48.3 to 43.5 nm (Figure 3). Among themodified products, the US-GR group exhibited the smallest distribution range (−39.8 to 29.2 nm), followed by the US group (−34.1 to 30.2 nm), and the GR group had the largest range (−39.6 to 38.2 nm). The thermal and mechanical effects of ultrasonic waves effectively disrupt the aggregates formed by antioxidant peptide components in aqueous solutions, leading to a reduction in their three-dimensional size distribution range (38). Additionally, the glycosylation reaction introduces more hydrophilic groups into the antioxidant peptide components, which reduces intermolecular forces and disrupts aggregate structures, resulting in glycosylated products with smaller particle sizes (2). This likely explains why the US-GR group exhibited the smallest distribution range. Overall, ultrasound plays a key role in preventing intermolecular interactions during the glycosylation of the British red kidney bean protein antioxidant peptide fractions (BHPs), thereby avoiding the formation of larger conjugates. Consequently, the resulting particles have a smaller and more uniform size distribution.

#### Intrinsic fluorescence spectrum

**Figure 4:**
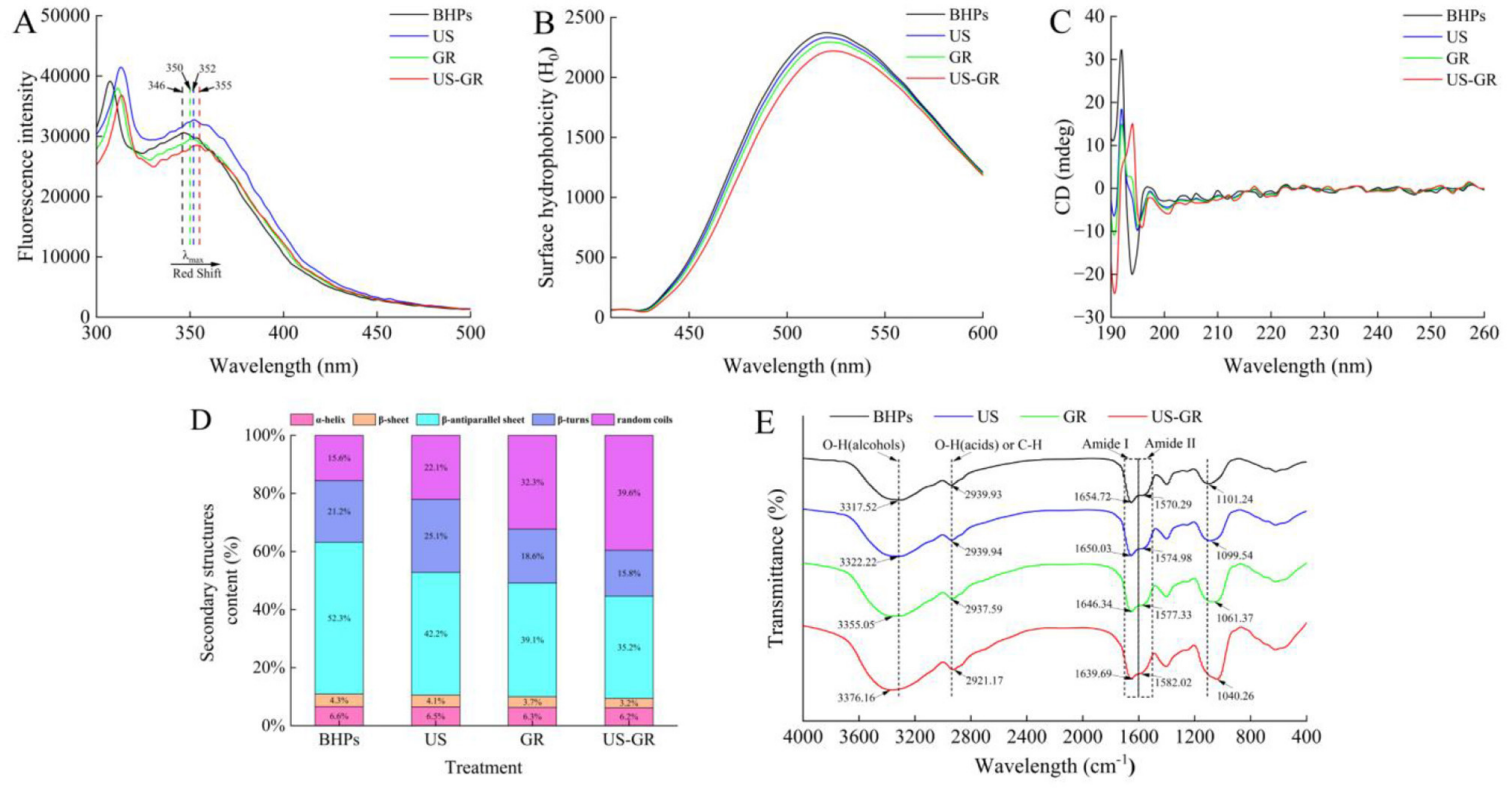
Changes in microstructure of the British red kidney bean protein antioxidant peptide fractions before and after ultrasound-assisted glycosylation modification, fluorescence absorption spectrum **(A)**, surface hydrophobicity (H0) **(B)**, circular dichroism spectra (CD) **(C)**, the relative content and proportion of secondary structures **(D)** and fourier transform infrared spectroscopy (FT-IR) **(E)**.

Tryptophan possesses fluorescence emission characteristics and is typically buried inside the protein structure. Changes in the maximum emission wavelength (λ _max_) of its intrinsic fluorescence spectrum can reflect alterations in the tertiary structure of proteins (39). Figure 4A shows that the λ _max_ of BHPs is located at 346 nm, while the λ _max_ of the US, GR, and US-GR groups are shifted to 352 nm, 350 nm, and 355 nm, respectively. All modified groups exhibit a red-shift compared to BHPs, with the US-GR treatment having the most significant effect on the tertiary structure of BHPs. Ultrasonic treatment (US) causes an increase in λ _max_, primarily due to the cavitation effect of ultrasound, which unfolds the structure of the antioxidant peptide components and exposes more chromophores. In contrast, glycosylation modification (GR) leads to a decrease in λ _max_, indicating that the glucose structure from glycosylation shields the chromophores (26). The US-GR treatment group has the smallest λ _max_, likely due to the dispersion of more glycosylation products into smaller fragments under the ultrasonic field, which subsequently crosslink to form aggregates. These aggregates embed the chromophores, resulting in reduced fluorescence intensity (40). These results suggest that ultrasonic treatment facilitates the unfolding of the British red kidney bean protein antioxidant peptide fractions structure, promoting glycosylation.

#### H_0_

In water-soluble proteins, hydrophobic residues are typically located within the interior of protein molecules. Surface hydrophobicity (H_0_) indicates the distribution of hydrophobic residues on the protein surface, which can reflect changes in the protein's higher-order structure (41). The order of surface hydrophobicity is BHPs, US, GR, and US-GR. After ultrasonic treatment and glycosylation, the hydrophobic residues of BHPs become embedded within the glycosylated products (Figure 4B). Similarly, glycosylation (GR) introduces hydrophilic hydroxyl groups from sugars onto the surface of BHPs, altering their microenvironment. Both treatments result in a decrease in surface hydrophobicity of BHPs (36, 42). In the US-GR treatment group, the combined effects of ultrasound and glycosylation facilitate the grafting of more hydrophilic groups onto the surface of the peptide components, which have been unfolded by the cavitation effect of ultrasound, further reducing surface hydrophobicity. These results are consistent with those of Feng Yuchao et al. (43).

#### CD

The α-helix structure of proteins typically shows a positive peak near 190 nm, and two negative peaks at 208 and 222 nm in circular dichroism spectra, while the β-sheet structure exhibits a negative peak around 216 nm and a positive peak in the range of 185–200 nm (44). As shown in Figure 4C, BHPs display α-helix characteristic peaks at 208 and 222 nm, and a negative peak at 216 nm, indicating the presence of both α-helix and β-sheet structures. Figure 4D reveals the relative content of different secondary structures, showing a downward trend in α-helix, β-sheet, β-antiparallel sheet, and β-turn contents in the US, GR, and US-GR treatment groups compared to BHPs. Conversely, the content of random coils increases. These changes suggest that the effects of US, GR, and US-GR treatments on the α-helix and β-sheet structures of BHPs are minimal, but their influence on β-antiparallel sheet, β-turn, and random coils is more significant. Among the treatments, the US-GR group exhibits the greatest changes in secondary structure content. In addition, it has been reported that random curls and β-turns are the main structural features embodying the biological activity of antioxidant peptides (45). Therefore, the increase in antioxidant activity of the modified products may be due to the different modification treatments that increased the content of irregular curls in the products. In glycosylation reactions, the carbonyl group of sugars covalently binds with free amino groups in the protein structure, leading to structural changes within the protein molecule (35). The cavitation effect of ultrasound disrupts intermolecular interactions, exposing more glycosylation sites (46). Furthermore, the modified products of BHPs show a tendency for α-helix, β-sheet, and β-antiparallel sheet structures to convert into β-turns and random coils. Another research found that ultrasound-assisted glycosylation modification led to a decrease in the β-fold content and an increase in the random curl content of rice proteins (29), which is similar to the trend of the results of this experiment.

Chen et al. (46) found that ultrasound-assisted glycosylation modification reduced the β-sheet content and increased random coils in rice protein, showing a similar trend.

#### FTIR

FTIR spectroscopy provides information on the vibrational states of chemical bonds in proteins and is commonly used to characterize the changes in functional groups after protein glycosylation modification (47). In the FTIR spectra, characteristic absorption peaks appear between 1,700 and 1,600 cm^−1^ and 1,600–1,500 cm^−1^, corresponding to the C=O stretching vibration of the amide I band and the N-H bending vibration of the amide II band (48). Compared to the BHPs at 1654.72 cm^−1^ and 1570.29 cm^−1^, the absorption peaks for the amide I band shift to longer wavelengths (red shift), and the absorption peaks for the amide II band shift to shorter wavelengths (blue shift) for thethree modified products, indicating that the secondary structure of BHPs has changed after treatment (Figure 4E). Among the treatments, the red and blue shifts are most significant in the US-GR group. This may be due to the mechanical and thermal effects of ultrasound cavitation, as well as the covalent bonding between the carbonyl group of glucose and the amino groups of BHPs, which reduces free amino groups and leads to a shift from an ordered to a disordered secondary structure, affecting the stretching vibrations of peptide bonds and hydrogen bond donors. This finding is consistent with the results of Abdelhedi et al. (49). At around 3317.52 cm^−1^, a broad peak and at 2939.93 cm^−1^, a secondary peak represent O-H and C-H stretching vibrations in BHPs, US, GR, and US-GR treatments (31). The blue shift in the 3317.52 cm^−1^ region for US-GR is larger than in BHPs, US, and GR, likely due to ultrasound promoting more glycosylation reactions between BHPs and potato reducing sugars, resulting in more hydroxyl groups in US-GR, consistent with the findings of Li et al. (41). The characteristic peaks in the 1180–950 cm^−1^ range, often referred to as the “sugar” band, reflect the covalent bonding between sugar molecules and proteins (36). Compared to BHPs, US, and GR, US-GR shows a higher peak absorption intensity at 1040.26 cm^−1^. This further supports the notion that ultrasound-assisted treatment promotes more glycosylation reactions between BHPs and potato reducing sugars, confirming that ultrasound facilitates the glycosylation reaction.

### Functional property analysis

#### Solubility

**Figure 5:**
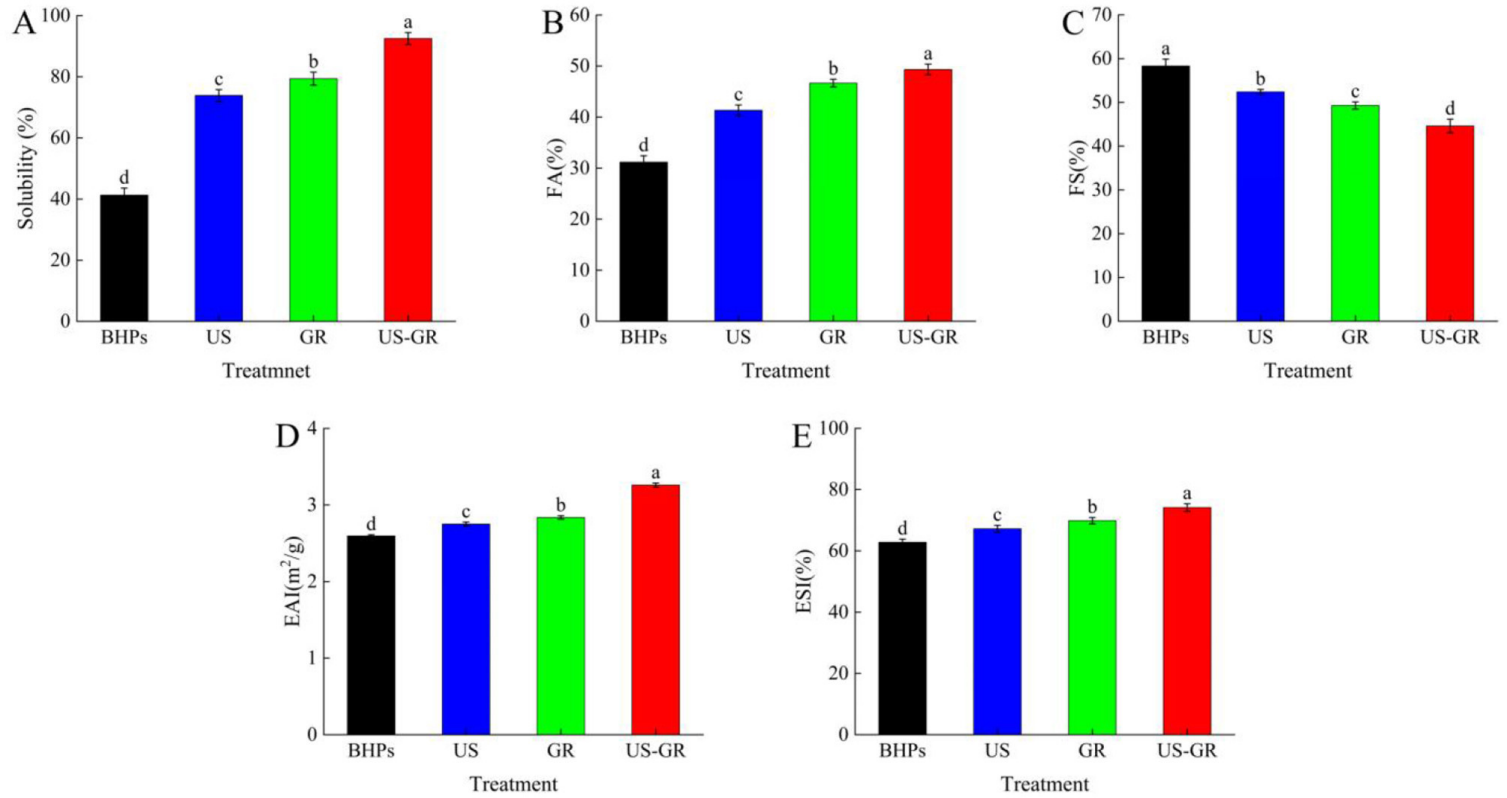
Changes in solubility **(A)**, foam properties (FA) **(B)**, foam stability (FS) **(C)**, emulsifying activity (EAI) **(D)** and emulsifying stability (ESI) **(E)** of British red kidney bean protein antioxidant peptide components before and after glycosylation modification. Different lowercase letters over column indicate significant differences (*p* < 0.05).

Solubility is an important property of proteins in the food industry, influencing functional characteristics such as emulsification, thickening, and gelation (44).As depicted in Figure 5A, the initial solubility of BHPs was 41.30%. After treatments with ultrasound (US), glycosylation reaction (GR), and a combination of US-GR, the solubility significantly increased to 73.89%, 79.39%, and 92.51%, respectively (*p* < 0.05). Ultrasound treatment enhanced solubility by modifying the surface chemical properties of the peptides, increasing the content of free amino and thiol groups while reducing surface hydrophobicity (31). The glycosylation reaction, which introduced hydrophilic groups such as hydroxyl groups from reducing sugars, further improved the solubility of the protein (16). Additionally, the US-GR treatment facilitated the incorporation of more hydrophilic groups into the peptides, further enhancing the solubility of the antioxidant peptides from British red kidney beans. These results suggest that US-GR is a highly effective method for improving the solubility of BHPs.

#### Foam properties

Foam activity (FA) refers to the ability of a solute to form bubbles in a solvent, while foam stability (FS) refers to the ability of the formed bubbles to remain stable under agitation or other disturbances (50). As shown in Figures 5B, C, the foam ability and foam stability of BHPs are 31.17% and 58.33%, respectively. After treatment with US, GR, and US-GR, foamability increased while foam stability decreased, showing significant differences (*p* < 0.05). Among them, the US-GR treatment exhibited the highest foamability (49.33%) and the lowest foam stability (44.61%). In the glycosylation reaction of antioxidant peptide components, the covalent bonding of sugars with peptide components improved the interfacial properties of the peptides (34). Meanwhile, ultrasound further enhanced the flexibility of the peptides, promoting adsorption at the air-solvent interface and increasing foamability. However, it also induced some aggregation of the peptide components, affecting foam stability (51, 52). Therefore, ultrasound-assisted glycosylation of BHPs improved foam activity while decreasing foam stability.

#### Emulsifying properties

Emulsifying properties are an important indicator of a protein's ability to adsorb at the oil/water interface, which can be evaluated by the emulsifying activity index (EAI) and emulsifying stability index (ESI) (52). The emulsifying activity (EAI) and emulsifying stability (ESI) of the modified British red kidney bean protein antioxidant peptide components (BHPs) were significantly improved (Figures 5D, E). This is related to the ultrasonic treatment that disperses the BHPs aggregates and reduces the aggregate size, thereby enhancing the interfacial adsorption capacity of BHPs (50). Meanwhile, the grafting of sugars may increase the hydration ability of BHPs, thus providing higher surface activity and adsorbability at the oil/water interface (53). Compared to BHPs (EAI = 2.60 m^2^/g; ESI = 62.82%), after US-GR treatment, both EAI and ESI increased by 25.38% and 18.05%, respectively, indicating that ultrasound-assisted glycosylation can further enhance the emulsifying properties of British red kidney bean protein peptides. Research shows that under the assistance of ultrasound, the degree of grafting increases, and the glycosylated products adopt a more open structure. The conjugates obtained can adsorb more tightly at the interface, helping to reduce the interfacial energy required for the oil/water interface and reduce droplet size, thereby improving emulsification ability (7). In conclusion, ultrasound-assisted glycosylation is an effective technique for improving the emulsifying properties of British red kidney bean protein peptides.

## Conclusion

This research primarily investigated the effects of ultrasound treatment on the antioxidant capacity, structure, and functional properties (foamability and emulsifying properties) of glycosylated products derived from the hydrolysis of potato starch and reduction-sugar glycosylation of the British red kidney bean protein antioxidant peptide fractions. Compared to the antioxidant peptide group, the ultrasound treatment group, glycosylation group, and ultrasound-assisted glycosylation group exhibited significantly enhanced antioxidant activity. The degree of intermolecular aggregation of the antioxidant peptides was reduced, and the spacing between them became more uniform. Both the free amino acid content and the degree of grafting indicated an improved glycosylation level. The molecular structure became more relaxed, exhibiting a higher degree of disorder. These findings suggest that ultrasound treatment facilitates structural transformation of the antioxidant peptides, promoting glycosylation reactions with reducing sugars. Additionally, ultrasound-assisted glycosylation significantly enhanced the foaming and emulsifying properties of the products. Meanwhile, in the results of this study, it was also shown that ultrasonic treatment had a positive effect on the functional properties of the British red kidney bean protein antioxidant peptide fractions, but the effect of ultrasonic treatment on the integrity of the peptide fractions is not yet clear. Some current studies have shown that ultrasonication leads to changes in peptide structure and activity, but whether or not a positive effect occurs depends greatly on the characteristics of the ultrasonication conditions. In the future, considering a systematic and complete elaboration of this process, the group will focus on the potential relationship between the enhancement of peptide antioxidant capacity and glycosylation degree by this technique and the conformational transformation in the future. This research provides technical support for enhancing the antioxidant capacity of the British red kidney bean protein antioxidant peptide fractions and offers a theoretical foundation for applying ultrasound-assisted glycosylation to improve the functional properties of bioactive peptides, promoted the application of ultrasonic technology in the modification of proteins or protein hydrolysates.

## Data Availability

The original contributions presented in the study are publicly available. This data can be found here: https://doi.org/10.6084/m9.figshare.29195408.v1.
